# Economy of Fertilizer Nitrogen through Organic Sources in Rain-Fed Rice-Legume Cropping Systems in West Bengal, India

**DOI:** 10.1100/tsw.2001.456

**Published:** 2001-12-11

**Authors:** A.M. Puste, S. Bandyopadhyay, D.K. Das

**Affiliations:** Department of Agronomy, Bidhan Chandra Krishi Viswavidyalaaaya, Nadia, West Bengal, India

**Keywords:** N fertilizer, farmyard manure, crop residues, green manure, Rhizobium inoculation, rain-fed rice-legume cropping systems, crop productivity

## Abstract

Field experiments were conducted at a farmers’ plot adjacent to the Regional Research Station, red and laterite zone, Sub-center Sekhampur (Birbhum district) of West Bengal, India, situated 23° 24' N latitude, 87° 24' E longitude, to study the effect of different bio- and organic sources of nutrients instead of total fertilizer N in terms of crop productivity in the sequence and building up of soil fertility. During the wet seasons of 1997 and 1998, 12 combinations of bio- and organic sources (crop residues, well decomposed cow dung, dhanicha as green manure) were substituted for 25–50% of N fertilizer applied on transplanted rice (Cv. IR 36). Subsequently, during the winters of 1997–1998 and 1998–1999, leguminous pulse crops like lentil (Lens culinaris [L.] Medic.), gram (Cicer arietinum L.) and lathyrus (Lathyrus sativus L.) were grown with and without inoculation of Rhizobium. Results revealed that the application of inorganic N in combination with organic sources exhibited a significant increase in rice yield (3.60–3.84 t ha) compared to the yield from sole application of N (3.19–3.26 t ha). The study showed that about 25% of total applied N was saved without significant yield reduction with simultaneous improvement of soil physical properties (pH, organic matter, available N, P, K, and CEC). Seed yield of pulses (lentil, gram, and lathyrus) were more pronounced in the treatment inoculated with Rhizobium, with a saving of 42.6–48.4 kg N ha. Therefore, the results suggest that the combined application of inorganic and organic N sources in a 75:25 ratio is a superior N-management practice with regards to crop yields as well as improvement of soil fertility.

## INTRODUCTION

The growing concern about environmental degradation, the shrinking of natural resources, and the urgent need to meet the food requirement of a burgeoning population are compelling scientists and policy makers to seriously examine alternatives to chemical agriculture. Current knowledge suggests that when a judicious combination of organic and inorganic sources of any management strategy is used to maintain soil fertility and stabilize and sustain the productivity in sequential cropping, especially in areas where land holdings are small and the resource base is weak, production and productivity reached a plateau. In India, N use efficiency continues to be very low, rarely exceeding 35%[1]. On-farm inputs like FYM, crop residues, and green manures are not only the storehouse of plant nutrients (macro- and microelements), but also help to improve the physico-chemical properties of soil. Introduction of pulse crop in the cropping sequence also provides an ample scope to improve soil fertility as well as fertilizer use efficiency, especially N in red and lateritic soils of the Indian subcontinent. Therefore, the integrated nutrient supply system simultaneously reduced the use of partly off-farm inputs, particularly nitrogenous fertilizer, without affecting the increase in crop yield. Keeping this in view, it is worthwhile to study the effect of integrated N management systems in rice-pulse cropping sequences on crop productivity vis-à-vis soil fertility in red lateritic soils of the Indian subtropics.

## EXPERIMENTAL METHODS AND PROCEDURES

The field experiment was carried out in red and lateritic acid soil (pH: 5.90) from June 1997 to March 1999, with the soil initially having 0.41% organic C, 0.84% organic matter, 11.8 C.mol.($P^+$) kg $ha^{-1}$ CEC. Available N, P, and K were 395.4, 17.4, and 159.6 kg $ha^{-1}$, respectively, at a farmers' field adjacent to the Regional Research Station, Sekhampur, under the red and laterite zone in West Bengal, India. The experiment was planned with 12 treatments, consisting of integrated nutrient management, by replacing different percentages of chemical fertilizers with FYM, well-rotten crop residues, dhanicha as green manure, or conc. neem oilcake on wet season transplanted rice (Cv. IR 36) from June to October of 1997 and 1998, using a randomized block design with 3 replications. During the winter seasons from November to March of 1997–1998 and 1998–1999, the experiment was continued in a split-plot design, wherein 12 residual effects of the treatments applied to preceding rice were considered as main plots ($F_1$: 100% of recommended N alone @ 60 kg $ha^{-1}$; $F_2$: 75% of recommended N alone; $F_3$: 50% of recommended N alone; $F_4$: 75% N + 3.0 t FYM $ha^{-1}$; $F_5$: 75% N + 3.65 t crop residues $ha^{-1}$; $F_6$: 75% N + 3.35 t green manure $ha^{-1}$; $F_7$: 75% N +0.5 t neem oilcake $ha^{-1}$; $F_8$: 50% N + 6.0 t FYM $ha^{-1}$; $F_9$: 50% N + 7.3 t crop residues $ha^{-1}$; $F_{10}$: 50% N + 6.7 t green manure $ha^{-1}$; $F_{11}$: 50% N +1.0 t neem oilcake $ha^{-1}$; $F_{12}$: control) and 3 winter pulses (lentil [*Lens culinaris* {L.} Medic.], gram [*Cicer arietinum* L.], and grass pea [*Lathyrus sativus* L.]) were sown in 3 subplots; the presence ($Rh_1$) and absence ($Rh_0$) of inoculation with *Rhizobium* sp. were considered in 2 sub-subplots. Among the winter pulses, lentil occupied the highest area in West Bengal, India. For comparison among the pulse crops in respect to grain yield, grain yield of gram and lathyrus was converted to equivalent yield of lentil, because all the pulse crops studied in this experiment are heterogeneous in characters in respect to their yield. Yield conversion of other pulses were done in terms of lentil equivalent by using this formula:

$$\text{Lentil grain equivalent} = \frac{\text{Total price of the crop to be compared (Rs.)}}{\text{Price of lentil kg}^{-1}\text{ (Rs.)}}$$

Soil samples were collected before the start of the experiment and after harvest of rice, lentil, gram, and grass pea in sequence. Collected soil samples were then air dried, ground, and passed through a 2-mm sieve. The pH, organic C, total N, CEC, available N, P, and K were determined by following the methods outlined in Jackson[2]. The recommended dose of N, $P_2O_5$, and $K_2O$ for rice was 60:30:30 kg $ha^{-1}$, of which 25 and 50% of N was applied through the different sources of organic matter. Adjusting this amount, the remaining part of N was applied in three equal splits, of which one third was applied as basal along with the total recommended amounts of $P_2O_5$ and $K_2O$, and rest in two splits of N at 25 and 50 DAT (days after transplanting) as top dressing. Different sources of organic matter were analysed on dry basis for determination of N content (FYM: 0.5%; well rotten crop residues: 0.41%; green manure (dhanicha): 0.65%; conc. bulk oil cake: 5.0%). The recommended doses of fertilizer for pulse crops was 20:40:0 kg N, $P_2O_5$, and $K_2O$ $ha^{-1}$. The entire amount of fertilizer was applied as basal (boosting dose) before sowing of pulse crops.

The seeds of lentil, gram, and lathyrus were inoculated with *Rhizobium leguminosarum* (ML-39), *Rhizobium* spp., and *R. leguminosarum* (MLa-15) respectively, and the dose of inoculum was 150 g $kg^{-1}$ of seed. The seeds of lentil, gram, and lathyrus were allowed to soak water overnight and in the morning; excess water was drained out as far as practical. The seeds were then thoroughly mixed with the respective strains of inoculum and air dried for half an hour in shade and sown accordingly.

The total rainfall received during the cropping season (June to March) was 1358.3 and 1416.7 mm in 68 and 72 wet days, of which maximum rainfall precipitated during June to September, during 1997 and 1998, respectively. The LTA (long-term average of 20 years) of rainfall during the crop season was 1309.94 mm in this subzone.

## RESULTS AND DISCUSSION

### *Effect of INM:* Yield of Wet Rice

Sole application of fertilizer N in different levels and substitution of fertilizer N by the application of organic N sources in different ratios showed a significant effect on the yield of rice, which might be due to favoured yield components, including test weight (Table 1). The highest grain (3.60–3.84 t $ha^{-1}$) and straw (4.02–4.12 t $ha^{-1}$) yield of rice was recorded in the treatment where a judicious combination of inorganic (75% fertilizer N) along with 3.35 t green manure $ha^{-1}$ was applied to rice crop. The lowest grain (1.52–1.64 to 2.56–2.68 t $ha^{-1}$) and straw (1.66–1.72 to 2.84–2.96 t $ha^{-1}$) yields were obtained in the treatments of the control ($F_{12}$) and even when 50–75% of the recommended doses of fertilizer N alone was applied ($F_2$ and $F_3$). The results further revealed that the decrement of yield was also recorded in $F_1$ treatment (100% N fertilizer), being in the range of 15.11–44.01 and 28.15–51.94% in grain and straw yield, respectively. The application of N with inorganic and organic sources on 50:50 basis resulted a yield loss to the tune of 19.79 and 24.27 in comparison to 75:25; these results are in agreement with the results obtained by Nambiar and Ghosh[3] and De Dutta et al.[4]. The study concluded with the findings that neither FYM nor crop

**TABLE 1**
**Yield Attributes and Yield of Wet-Season Rice**

| | '000 Grain Weight (g) | | Grain Yield (t ha$^{-1}$) | | Straw Yield (t ha$^{-1}$) | |
|---|---|---|---|---|---|---|
| Treatments | 1997 | 1998 | 1997 | 1998 | 1997 | 1998 |
| $F_1$: 100% N alone (@ 60 kg ha$^{-1}$) | 21.12 | 21.46 | 3.19 | 3.26 | 3.16 | 3.22 |
| $F_2$: 75% N alone | 20.45 | 20.62 | 2.56 | 2.68 | 2.84 | 2.96 |
| $F_3$: 50% N alone | 19.05 | 19.16 | 2.08 | 2.15 | 1.86 | 1.98 |
| $F_4$: 75% N + 3.0 t FYM ha$^{-1}$ | 22.96 | 23.12 | 3.52 | 3.78 | 3.92 | 4.06 |
| $F_5$: 75% N + 3.65 t crop residues ha$^{-1}$ | 22.82 | 22.96 | 3.43 | 3.72 | 3.78 | 3.92 |
| $F_6$: 75% N+ 3.35 t green manure ha$^{-1}$ | 23.18 | 23.32 | 3.60 | 3.84 | 4.02 | 4.12 |
| $F_7$: 75% N + 0.5 t neem oilcake ha$^{-1}$ | 22.68 | 22.82 | 3.56 | 3.72 | 3.59 | 3.74 |
| $F_8$: 50% N + 6.0 t FYM ha$^{-1}$ | 21.85 | 21.92 | 3.02 | 3.14 | 3.11 | 3.21 |
| $F_9$: 50% N + 7.3 t crop residues ha$^{-1}$ | 21.58 | 21.62 | 2.96 | 3.08 | 3.02 | 3.12 |
| $F_{10}$: 50% N + 6.7 t green manure ha$^{-1}$ | 22.06 | 22.18 | 3.12 | 3.26 | 3.21 | 3.38 |
| $F_{11}$: 50% N + 1.0 t neem oilcake ha$^{-1}$ | 21.42 | 21.56 | 3.18 | 3.28 | 3.08 | 3.16 |
| $F_{12}$: control | 18.56 | 18.62 | 1.64 | 1.52 | 1.72 | 1.66 |
| CD ($p$ = 0.05) | 2.54 | 2.23 | 0.78 | 0.62 | 0.62 | 0.48 |

residues with high quality could substitute 50% of the recommended dose of chemical fertilizer in wet-season rice[5,6].

## Residual Effect of INM of Wet Rice on Pulses

Grain yield of pulses were significantly influenced by the residual INM effect, which was adopted in the preceding rice crop during the wet season. The residual effect of integrated nutrient management was reflected more in the treatments on $F_8$ to $F_{11}$, where all the pulse crops produced highest lentil equivalent (Table 2). The production was rather more evident in the second year of the experiment (0.894–1.168 t ha$^{-1}$) than in the initial year (0.812–0.843 t ha$^{-1}$). Treatment $F_{10}$ (50% fertilizer N + 6.7 t green manure ha$^{-1}$) dominated high among the treatments in which 50% of N was substituted by organic sources and signified more by about 60.88–140.33% than that of $F_1$ (100% N fertilizer alone) or $F_2$, $F_3$, etc. This was thus mainly the result of organic matter, which had a lasting effect, particularly on soil productivity[7]. Though the highest yield of rice was obtained with 100% of the recommended dose of NPK, but following winter, maximum lentil yields were recorded from the plots which were previously treated with 50% NPK + FYM[8]. It is rather more important particularly in a coarse-textured soil; considerable improvement may definitely be achieved due to the addition of large quantity of bulk manures with the soil. This has a tremendous potential for holding up more residual moisture as well as nutrients within soil root zone depth.

## Individual Pulse Crop

Pulse crops statistically differed among themselves in respect to the lentil equivalent yield (Table 2). Among the three, gram recorded maximum (0.774–0.858 t ha$^{-1}$) and was followed by lentil (0.721–0.791 t ha$^{-1}$) and grass pea (0.532–0.614 t ha$^{-1}$). This might be due to the fact that gram showed highest efficiency towards the utilization of residual soil moisture compared to lentil and grass pea in medium land situation in this subtropical region[9].

## *Effect of* Rhizobium

The lentil equivalent yield of all pulse crops were also influenced significantly by inoculation of *Rhizobium* culture with the seeds prior to sowing of the crop as compared to the absence of inoculation (Table 2). This has a positive effect for building up of additional soil N as well as boosting the productivity of such crops. By and large, inoculation with *Rhizobium* spp. enhanced pulse yield by 11.58–17.75%. From the experiment, it is quite justified that seed yield of pulses (lentil, gram, and lathyrus) inoculated with *Rhizobium* culture was more pronounced, a saving to the tune of 45.5 kg N (42.6 to 48.4 kg N) for the succeeding crops per hectare stored in the soil, which is a costly input in the subtropics. This finding is corroborated with the observations made by Bhattacharya and Sen[10].

## Soil Fertility Status

Fertility status, including the physical characteristics of such soil, significantly influenced the improvement of soil health over years of cropping sequence in this subtropical region. In keeping sustainability in crop productivity, the role of organic matter had a magnificent importance, particularly in such coarse-textured, less humus, and comparatively less productive soil. All the characteristics of soil analyzed after pulse are presented in Table 3.

**TABLE 2**
**Seed Yield of Winter Pulse Crops in Terms of Lentil Grain Equivalent**

| | Seed Yield of Pulses (t ha$^{-1}$) | | |
|---|---|---|---|
| **Treatment** | **1997–1998** | **1998–1999** | **Mean** |
| **Residual effect of wet rice treatments** | | | |
| $F_1$: 100% N alone @ 60 kg ha$^{-1}$ | 0.524 | 0.486 | 0.505 |
| $F_2$: 75% N alone | 0.465 | 0.453 | 0.459 |
| $F_3$: 50% N alone | 0.402 | 0.386 | 0.394 |
| $F_4$: 75% N + 3.0 t FYM ha$^{-1}$ | 0.796 | 0.826 | 0.811 |
| $F_5$: 75% N + 3.65 t crop residues ha$^{-1}$ | 0.780 | 0.818 | 0.799 |
| $F_6$: 75% N+ 3.35 t green manure ha$^{-1}$ | 0.816 | 0.938 | 0.877 |
| $F_7$: 75% N + 0.5 t neem oilcake ha$^{-1}$ | 0.722 | 0.784 | 0.753 |
| $F_8$: 50% N + 6.0 t FYM ha$^{-1}$ | 0.834 | 1.069 | 0.951 |
| $F_9$: 50% N + 7.3 t crop residues ha$^{-1}$ | 0.839 | 0.987 | 0.908 |
| $F_{10}$: 50% N + 6.7 t green manure ha$^{-1}$ | 0.843 | 1.168 | 1.006 |
| $F_{11}$: 50% N + 1.0 t neem oilcake ha$^{-1}$ | 0.812 | 0.894 | 0.853 |
| $F_{12}$: control | 0.286 | 0.244 | 0.265 |
| CD ($p$ = 0.05) | 0.071 | 0.083 | —- |
| **Pulse crops** | | | |
| Lentil | 0.721 | 0.791 | 0.756 |
| Gram | 0.774 | 0.858 | 0.816 |
| Lathyrus | 0.532 | 0.614 | 0.573 |
| CD ($p$ = 0.05) | 0.042 | 0.036 | —- |
| **Effect of *Rhizobium*** | | | |
| $Rh_0$ (control) | 0.639 | 0.693 | 0.666 |
| $Rh_1$ (*Rhizobium* inoculation) | 0.713 | 0.816 | 0.764 |
| CD ($p$ = 0.05) | 0.038 | 0.046 | —- |

## Residual Effect of INM on Wet Season Rice

The substitution of 25–50% of chemical N fertilizer by different sources of organic matter of different quantities remarkably influenced the soil characteristics (pH, organic matter, available N, P, and K, and CEC value of the soil). These values were more pronounced, particularly when a bulk quantity of organic matter (6.7 t of green manure ha$^{-1}$) was added with the soil in treatment $F_{10}$ (Table 3). Though the pH value of such soil did not show any statistical difference among the treatments, organic matter (1.32%), available N (704.6 kg ha$^{-1}$), P (22.7 kg ha$^{-1}$), K (168.9 kg ha$^{-1}$), and CEC (14.7 C.mol.[$P^+$]kg ha$^{-1}$) gained enough in the treatments $T_4$ to $T_7$, where 25% of chemical N fertilizer was substituted by various organic sources, to suggest that the gains might be due to the enrichment for sufficient humus with the soil. The treatments containing inorganic N fertilizers alone ($F_1$ to $F_3$) exhibited least effect on these characters. Almost all the treatments related to 50% N substitution by means of various organic sources ($T_8$ to $T_{11}$) were statistically at par in respect to soil characteristics, assuming that a similar effect may be sustained within the soil stratum over this application.

## Pulse Crops

All the characteristics related to the soil improvement were statistically at par when comparing among the pulse groups of the experiment, probably due to the similar trend of biomass production including nodulation in their root system (Table 3). All of the parameters for gram were highest, while lentil produced the lowest values.

### *Effect of* Rhizobium

Soil physico-chemical properties like organic matter and available N, P, and K were significantly different in inoculation and noninoculation of pulse seeds before sowing (Table 3). CEC value of such soil was at par, being highest value with the inoculation of *Rhizobium* culture (13.87 C.mol.[$P^+$]kg ha$^{-1}$). Significant increased value of organic C (0.62%), organic matter (1.07%), and available N, P, and K (549.6, 20.2, and 161.8 kg ha$^{-1}$) were favoured with *Rhizobium* inoculation and such soil fertility was eventually attained after pulse in the rice-pulse cropping system.

**TABLE 3**
**Soil Fertility Status After Harvesting of Last Pulse Crops**

| Treatment | pH | Org. matter (%) | Av. N (kg ha$^{-1}$) | Av. P (kg ha$^{-1}$) | Av. K (kg ha$^{-1}$) | CEC C.mol. ($P^+$) kg$^{-1}$ |
|---|---|---|---|---|---|---|
| **Residual effect of wet rice treatments** | | | | | | |
| $F_1$: 100% N alone @ 60 kg ha$^{-1}$ | 5.78 | 0.84 | 421.3 | 16.4 | 152.4 | 12.7 |
| $F_2$: 75% N alone | 5.80 | 0.81 | 392.4 | 16.2 | 152.1 | 12.9 |
| $F_3$: 50% N alone | 5.80 | 0.77 | 352.6 | 16.1 | 150.3 | 12.1 |
| $F_4$: 75% N + 3.0 t FYM ha$^{-1}$ | 5.82 | 1.07 | 560.4 | 18.9 | 156.3 | 13.9 |
| $F_5$: 75% N, + 3.65 t crop residues ha$^{-1}$ | 5.75 | 1.01 | 522.6 | 18.3 | 155.6 | 13.3 |
| $F_6$: 75% N+ 3.35 t green manure ha$^{-1}$ | 5.80 | 1.12 | 576.3 | 19.7 | 157.9 | 14.2 |
| $F_7$: 75% N + 0.5 t neem oilcake ha$^{-1}$ | 5.78 | 1.00 | 504.6 | 17.5 | 155.7 | 13.1 |
| $F_8$: 50% N + 6.0 t FYM ha$^{-1}$ | 5.82 | 1.24 | 681.2 | 21.6 | 167.5 | 14.2 |
| $F_9$: 50% N + 7.3 t crop residues ha$^{-1}$ | 5.80 | 1.20 | 656.4 | 20.6 | 162.4 | 13.8 |
| $F_{10}$: 50% N + 6.7 t green manure ha$^{-1}$ | 5.74 | 1.32 | 704.6 | 22.7 | 168.9 | 14.7 |
| $F_{11}$: 50% N + 1.0 t neem oilcake ha$^{-1}$ | 5.83 | 1.19 | 630.6 | 19.3 | 163.7 | 13.4 |
| $F_{12}$: control | 5.86 | 0.74 | 326.5 | 14.9 | 150.2 | 12.1 |
| CD ($p$ = 0.05) | N.S. | 0.15 | 78.4 | 1.86 | 4.83 | 0.96 |
| **Pulse crops** | | | | | | |
| Lentil | 5.81 | 1.01 | 516.7 | 17.7 | 154.7 | 12.91 |
| Gram | 5.76 | 1.05 | 541.6 | 19.2 | 160.6 | 13.43 |
| Lathyrus | 5.83 | 1.01 | 524.1 | 18.7 | 158.2 | 13.76 |
| CD ($p$ = 0.05) | N.S. | N.S. | N.S. | N.S. | N.S. | N.S. |
| **Effect of *Rhizobium*** | | | | | | |
| $Rh_0$ (control) | 5.88 | 0.98 | 504.1 | 16.8 | 153.9 | 12.86 |
| $Rh_1$ (*Rhizobium* inoculation) | 5.72 | 1.07 | 549.6 | 20.2 | 161.8 | 13.87 |
| CD ($p$ = 0.05) | N.S. | 0.052 | 39.7 | 2.17 | 5.08 | 1.01 |

*Note:* N.S. = Not significant.

## CONCLUSION

From the present study it may be concluded that 25% of the recommended dose of N from chemical fertilizers instead of 100% can effectively be substituted for any of the organic sources (green manure, crop residues, FYM, oilcake), which not only produced the highest yields of rice but also the favourable residual effects brought about significantly in a better way from successive pulse crops. The integrated nutrient management system along with a microbiological approach (*Rhizobium* culture) improved the soil physical and chemical properties considerably and thereby increased the total productivity of the rice-pulse cropping system on a sustained basis. This is more important for enrichment of such rain-fed, medium coarse-textured soils of the lateritic soil belt of this Indian subtropical region, where the soil is really poor in respect to water as well as nutrient-holding capacity as a whole.

## ACKNOWLEDGEMENTS

The authors are thankful to the Department of Agriculture of the Government of West Bengal for providing all sorts of help during the period of investigation.

| | | |
|---|---|---|
| Received: | July | 10, 2001 |
| Revised: | November | 12, 2001 |
| Accepted: | November | 15, 2001 |
| Published: | December | 11, 2001 |
